# Why could meditation practice help promote mental health and well-being in aging?

**DOI:** 10.1186/s13195-018-0388-5

**Published:** 2018-06-22

**Authors:** Gaël Chételat, Antoine Lutz, Eider Arenaza-Urquijo, Fabienne Collette, Olga Klimecki, Natalie Marchant

**Affiliations:** 1 Inserm UMR-S U1237, Université de Caen-Normandie, GIP Cyceron, Bd Henri Becquerel – BP 5229, 14074 Caen Cedex, France; 2 Lyon Neuroscience Research Center INSERM U1028, CNRS UMR5292, Lyon 1 University, Lyon, France; 3 GIGA-CRC in Vivo Imaging, University of Liège, Liège, Belgium; 4 Psychology and Neuroscience of Cognition, University of Liège, Liège, Belgium; 5 Fund for Scientific Research FNRS, 1000 Brussels, Belgium; 6 Swiss Center for Affective Sciences, Department of Medicine and Department of Psychology, University of Geneva, Geneva, Switzerland; 7 University College London, Division of Psychiatry, 6th Floor Maple House, 149 Tottenham Court Road, London, W1T 7NF UK

**Keywords:** Mindfulness, Lifestyle, Non-pharmacological intervention, Older adults, Aging, Well-being, Brain reserve, Maintenance, Neuroimaging, Meditation

## Abstract

Psycho-affective states or traits such as stress, depression, anxiety and neuroticism are known to affect sleep, cognition and mental health and well-being in aging populations and to be associated with increased risk for Alzheimer’s disease (AD). Mental training for stress reduction and emotional and attentional regulation through meditation practice might help reduce these adverse factors. So far, studies on the impact of meditation practice on brain and cognition in aging are scarce and have limitations but the findings are encouraging, showing a positive effect of meditation training on cognition, especially on attention and memory, and on brain structure and function especially in frontal and limbic structures and insula. In line with this, we showed in a pilot study that gray matter volume and/or glucose metabolism was higher in six older adult expert meditators compared to 67 age-matched controls in the prefrontal, anterior and posterior cingulate cortex, insula and temporo-parietal junction. These preliminary findings are important in the context of reserve and brain maintenance as they suggest that long-term meditation practice might help preserve brain structure and function from progressive age-related decline. Further studies are needed to confirm these results with larger samples and in randomized controlled trials and to investigate the mechanisms underlying these meditation-related effects. The European Commission-funded project Silver Santé Study will address these challenges by studying 316 older adults including 30 expert meditators and 286 meditation-naïve participants (either cognitively normal or with subjective cognitive decline). Two randomized controlled trials will be conducted to assess the effects of 2-month and 18-month meditation, English learning or health education training programs (versus a passive control) on behavioral, sleep, blood sampling and neuroimaging measures. This European research initiative illustrates the progressive awareness of the benefit of such non-pharmacological approaches in the prevention of dementia and the relevance of taking into account the psycho-affective dimension in endeavoring to improve mental health and well-being of older adults.

## Introduction

**Fig. 1:**
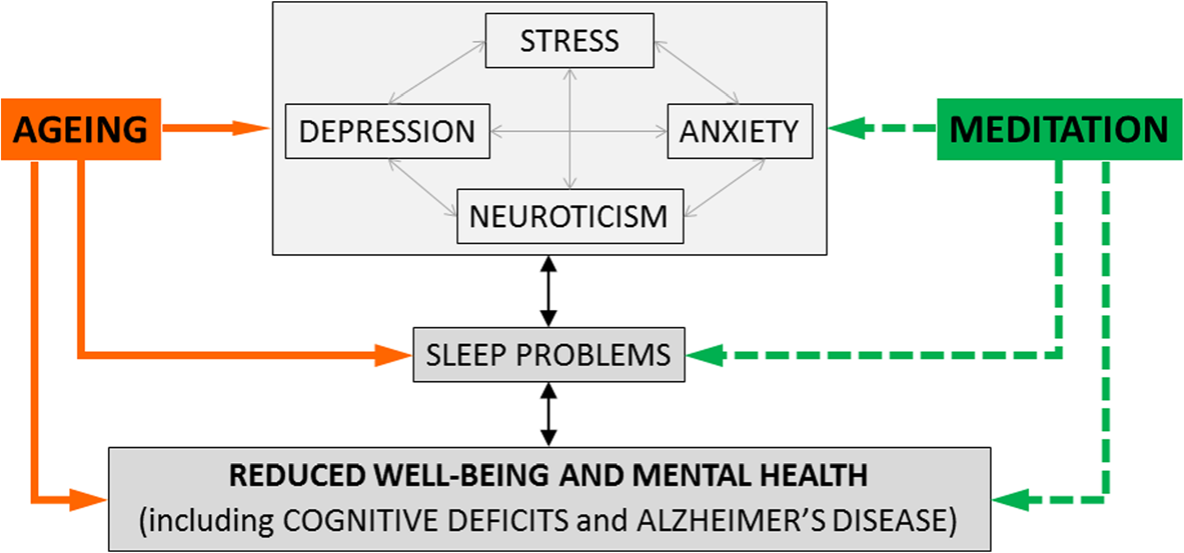
The effects of psycho-affective states or traits (stress, depression, anxiety, neuroticism) on mental health and well-being in aging populations and risks for dementia. Meditation training might help reduce these adverse psycho-affective states or traits, and thereby improve sleep, cognition, and mental health and well-being in aging and reduce the risk, or delay the onset, of Alzheimer’s disease

As our population is aging, with a considerable increase in both the number and the proportion of people aged 60 years or over, increasing healthy life years is a priority. Up to 50% of older adults have sleep disturbances [1], 10 to 15% are affected by late-life depression [2], and almost 10% have dementia. There is no curative treatment for dementia but it is now acknowledged that several lifestyle factors (e.g. diet, cognitive and physical activities) have an impact on brain aging and the development of dementia. Moreover, about one-third of Alzheimer’s disease (AD) cases would be due to modifiable risk factors (such as smoking, hypertension and smoking) [3] and a risk factor reduction of 25% could potentially prevent up to three million AD cases worldwide [4]. Some psycho-affective states or traits are also potentially modifiable risk factors. Thus, each depressive symptom increases dementia risk by approximately 20% [5], stress has a detrimental effect on hippocampus integrity [6], and neuroticism and anxiety are associated with an increased cumulative incidence of dementia [7, 8]. These conditions are often associated with sleep difficulties, themselves associated with AD-related pathological processes [9, 10]. Depression, anxiety, and insomnia, but also subclinical conditions such as stress, worry, sleep disturbances and cognitive decline, deteriorate well-being and mental health of older adults, have a negative impact on brain structure and function, and increase the risk of dementia (Fig. 1), and thus represent a considerable burden for the elderly, their family members and caregivers, and society at large.

Mental training for stress reduction and attention and emotion regulation through meditation practice might help reduce cognitive decline and these adverse psycho-affective factors [11, 12]. This training might in turn result in reduced risk or delayed onset of dementia, and more generally in improved quality of life of aging populations [13] and an increase in healthy life years (Fig. 1).

## Main text

### Brief definition of meditation

The term “meditation” refers to a broad variety of practices. Here we define meditation as a family of complex emotional and attentional regulatory strategies developed for various ends, including the cultivation of well-being and emotional balance [14]. Secular mindfulness-based meditation is most often used in scientific research. Mindfulness, or attentive presence, consists of cultivating a vigilant awareness of one’s own thoughts, actions, emotions and motivations and directly target attention and emotion regulation ability, which is particularly important in the context of aging.

### Previous studies on meditation in aging populations

Promising evidence exists in older adults that meditation reduces stress, anxiety, depression, insomnia, feelings of loneliness and social exclusion [12, 13], and cardiovascular risk factors [15]. Studies on the impact of meditation on brain and cognition in aging are scarce but the findings are encouraging. They have shown, for example, a positive effect of meditation training on cognition, especially on attention and memory [16], known to be particularly sensitive to aging and AD. Yet, further studies are needed, including randomized control trials using large samples to confirm that meditation training improves cognition in older adults as previous studies have limitations [17]. Meditation training has also been associated with increased telomerase activity in blood cells [18], itself associated with health and longevity. As for the effects on the brain, studies conducted in young and middle-aged adults have shown meditation-related increases in brain structure and function, especially in frontal and limbic structures, as well as in the insula [19, 20]. These results are particularly interesting in the context of aging as age-related decreases in cerebral volume and glucose metabolism are known to predominate in the frontal and anterior cingulate cortex and insula. One previous neuroimaging study assessed how meditation practice modulates brain aging from 24 to 77 years old [21]. It showed that gray matter volume reduction with age was less marked in a group of meditation practitioners than in a group of non-meditators [21]. In our lab, we conducted a pilot study comparing magnetic resonance imaging and fluorodesoxyglucose-positron emission tomography (PET) data in six older adult expert Buddhist meditators (>  10,000 h of practice) versus 67 older adult controls. We showed that gray matter volume and/or glucose metabolism was higher in expert meditators than controls in the prefrontal, anterior and posterior cingulate cortex, insula and temporo-parietal junction [22]. Interestingly, most of these regions also showed the strongest age-related decrease from 20 to 87 years in a cohort of 186 controls [22], and the temporo-parietal and posterior cingulate cortex are known as being the most sensitive brain regions to early AD-related glucose metabolism changes [23]. To date, no other study has assessed the effect of meditation on brain glucose metabolism. The findings of this first study are potentially important in the context of reserve, brain maintenance and prevention. Pending replication in a larger sample, they suggest that long-term meditation might help preserve brain structure and function from progressive age-related decline. This could eventually lead to increased brain and cognitive reserve, and reduced risk or delayed onset of dementia in general and AD in particular.

### Future directions and the on-going European Medit-aging project

Further studies with larger cohorts of older adult expert meditators and longitudinal studies assessing the effects of meditation on meditation-naïve older individuals in randomized controlled trials are warranted. Moreover, future research is needed to investigate the mechanisms underlying the effects of meditation, especially in the context of aging and AD.

The European commission has funded a project called Medit-Aging designed to specifically address these challenges (public name Silver Santé Study; www.silversantestudy.eu). The consortium includes ten partners in six European countries. Two randomized controlled trials sponsored by Inserm will be conducted. First, 160 patients with subjective cognitive decline will be randomized to a 2-month mindfulness-based meditation training or a health education program, and behavioral (cognitive, lifestyle and well-being) and blood measures will be performed before and after the intervention as well as 4 months later. Second, 126 cognitively normal older adults will be randomized in three arms (18-month meditation, an English learning program or a passive control condition), and behavioral, blood sampling, sleep and neuroimaging measures will be performed before and after the 18-month intervention. The meditation intervention will teach mindfulness, kindness and compassion-based meditations. Moreover, 30 long-term older adult meditators will be included to help understand the mechanisms.

## Conclusions

Prevention is a priority to reduce the burden of dementia. Promoting changes in lifestyle via non-pharmacological interventions is our best hope toward prevention. Psycho-affective states or traits such as stress, anxiety, depression and neuroticism are known risk factors for dementia, yet they are rarely targeted in preventive non-pharmacological interventions. Indirect evidence, or direct but preliminary reports, indicate that meditation training for stress reduction and emotional and attentional regulation would benefit mental and brain health and well-being in aging populations. Collaborative efforts toward large-scale randomized controlled clinical trials are needed to investigate this prospect, which has led to the launch of the European project Silver Santé Study.
